# Correlation Between PAPP-A Levels Determined During the First Trimester and Birth Weight at Full-Term

**DOI:** 10.1007/s43032-023-01270-4

**Published:** 2023-05-26

**Authors:** E. M. Turrado Sánchez, V. De Miguel Sánchez, M. Macía Cortiñas

**Affiliations:** Obstetrics and Gynecology, Santiago de Compostela University Clinical Hospital, A Coruña, Spain

**Keywords:** First-trimester combined screening (FTCS), Intrauterine restricted growth (IURG), Large for gestational age (LGE), Pregnancy-associated plasma protein-A (PAPP-a), Percentile (p)

## Abstract

Foetal birth weight is an important determinant of perinatal health. For this reason, various methods have been investigated for estimating this weight during pregnancy. The aim of this study is to evaluate the possible relationship between full-term birth weight and pregnancy-associated plasma protein-A (PAPP-A) levels determined during the first trimester as part of combined screening for aneuploidy carried out in pregnant women. We carried out a single-centre study including pregnant women who were being followed up by the Obstetrics Service Care Units of the XXI de Santiago de Compostela e Barbanza Foundation, who gave birth from March 1, 2015, to March 1, 2017, and who had undergone their first-trimester combined chromosomopathy screening. The sample included a total of 2794 women. We found a significant correlation between MoM PAPP-A and foetal birth weight. When MoM PAPP-A was measured at extremely low levels (< 0.3) during the first trimester, the OR for giving birth to a foetus with weight < p10, adjusting for gestational age and sex, was 2.74. For low levels of MoM PAPP-A (0.3–0.44), the OR was 1.52. With regard to the value of MOM PAPP-A levels as a predictor of foetal macrosomia, a correlation could be observed with elevated levels, although this was not statistically significant. PAPP-A determined during the first trimester acts as a predictor of foetal weight at term as well as for foetal growth disorders.

## Introduction

Foetal growth is determined by the interaction between foetal growth potential (predetermined by the foetus itself) and foetal, placental, and maternal factors. A total of 30–50% of foetal weight is determined by genetic factors, whilst the remainder is determined by environmental factors [1]. Foetal weight is an indicator of perinatal mortality, as it allows us to determine both the health status of the foetus as well as to predict certain complications in the mother [2].

Firstly, the term large (foetus) for gestational age (LGE) (also known as *macrosomia*) is used to refer to foetuses whereby the estimated foetal weight (EFW) is greater than the 90th percentile (p90) for their gestational age [3, 4]. At birth weights above 4000 g, the rates of both maternal and neonatal complications increase. The main risk to the mother associated with macrosomia is an increased risk of Caesarean section and an increased risk of perineal lesions (2- to threefold increased risk of third- and fourth-degree tears), particularly if labour is complicated by shoulder dystocia (SD) and/or post-partum haemorrhage [5, 6]. Mortality is higher in foetuses with macrosomia. These children also have an increased risk of scoring an Apgar < 3 at the first minute of life, having respiratory distress syndrome, and experiencing meconium aspiration [7].

On the other hand, foetuses that are small for gestational age fall below the 10th growth percentile (p10), adjusted for their gestational age and sex; however, many other definitions have been proposed that apply different criteria. When a foetus is small, it is important to distinguish between whether it is a constitutionally small foetus given the gestational age (SGA) or if there is intrauterine growth restriction (IUGR). An SGA foetus is defined as a foetus weighing between p3–10, adjusting for gestational age (GA), due to constitutional factors including height, weight, ethnicity, and parity of the mother. These foetuses can be diagnosed using ultrasound, which does not reveal any abnormalities on Doppler imaging [8]. This group of children has no increased morbidity or mortality compared to foetuses of normal weight [9]. By contrast, foetuses with IUGR appear on ultrasound as being below p3 or else below p10 with abnormalities seen on Doppler imaging [8, 10–13]. This group is associated with a higher risk of adverse perinatal outcomes, such as intrauterine foetal death, neonatal morbidity or mortality, having a neuro-developmental disorder [14–17], as well as presenting with a greater frequency of certain pathologies in adult life (e.g. hypertension, hyperlipidaemia, and diabetes mellitus) [18–21] as compared to foetuses showing normal growth [17].

Plasma protein-A is produced in the human placenta (PAPP-A) and has a regulatory effect on insulin-like growth factor (IGF) concentration by means of proteolysis of the insulin-like growth factor binding protein (IGFBP) [22–25]. IGF acts locally to promote mitosis and cellular differentiation; it has effects on both embryogenesis and the regulation of foetal and placental growth [26]. As a consequence of this latter function, PAPP-A is considered a regulator of cell growth and proliferation.

There are numerous publications in the literature that describe relationships between a variation in PAPP-A levels and foetal complications. One of these relationships describes how low PAPP-A levels are associated with hypertensive states during pregnancy [27]. This relationship is also broadly applied in clinical practice as part of protocols that predict preeclampsia in the first trimester, whereby this risk is considered to be high if the PAPP-A value is less than 0.4 times the median value (TMV) [28].

Subsequent studies have been carried out on low or extremely low levels of PAPP-A and its associations with other pathologies such as intrauterine growth restriction [29], abortion [30], and gestational diabetes [31], but these relationships, unlike those mentioned above, have not been translated into clinical practice, and further studies are required in the field.

## Objectives

The aim of this study is to evaluate the possible relationship between full-term birth weight and PAPP-A levels determined during the first trimester carried out as an integral part of combined screening for aneuploidy.

## Materials and Methods

This single-centre study included pregnant women who were being followed up by the Obstetrics Service Care Units of the XXI de Santiago de Compostela e Barbanza Foundation, who met the inclusion and exclusion criteria as described in the following paragraphs, and who consented to be included in the study and signed the corresponding informed consent form once they had been provided with the relevant study protocol information sheet and a discussion had been held to explain the information sheet.

The scope of the study included pregnant women over the age of 18 who gave birth between March 1, 2015, and March 1, 2017, and who underwent first-trimester combined screening for chromosomopathies in our hospital.

The inclusion criteria were pregnant women (over 18 years of age) undergoing screening for trisomy 21 and 18 during their first trimester by means of PAPP-A testing of blood at the Santiago de Compostela University Hospital Complex between 11 + 0 and 13 + 6 weeks of gestation, who consented to be included in the study, and who signed the informed consent form for participation in the study once they had been provided with a patient information sheet and this information had been adequately explained to the patient.

The exclusion criteria were multiple pregnancies; incomplete data; abnormal foetal karyotype; previous spontaneous abortion.

During this period, there were 5273 births at our centre. Of these, 4970 births were from singleton pregnancies; 294 were from twin pregnancies; 9 births were from triplet pregnancies. Of the 4970 women with singleton pregnancies, full study data could not be obtained for 734 women. Of the remaining 4236 women, 2794 of them consented to participate in the study by signing the informed consent form.

This population has been selected for the study in order to evaluate the study objectives.

For the data analysis, an epidemiological, observational, analytical, longitudinal, and prospective study was designed. Once permission had been obtained from the Santiago-Lugo Research Ethics Committee (“CEI-SL”), dated February 24, 2015, with registration reference 2015/049, epidemiological and clinical data were obtained by reviewing each patient’s medical records as stored in IANUS (a platform for systems integration and storing of medical information, “Indra”). Clinical data were compiled and coded into the dedicated study Data Collection Logbook (DCL), ensuring patient identities were protected. The collected data could only be accessed by the members of the research team and the health authorities, all of whom were under an obligation to ensure confidentiality.

The project was implemented in accordance with the Declaration of Helsinki (1964) of the World Medical Association as well as ratifications made by subsequent assemblies (in Tokyo, Venice, Hong Kong, and South Africa) on the ethical principles for medical research carried out in humans, as well as Order SCO/256/2007 of February 5, 2007, laying down the principles and detailed guidelines of Good Clinical Practice and the Convention on Human Rights and Biomedicine, agreed in Oviedo on April 4, 1997, as well as subsequent revisions to this order.

In order to analyse the data collected during this study, a descriptive analysis was first carried out of the variables obtained from the patient’s clinical records; this analysis considered the epidemiological and clinical characteristics of the study group.

Continuous variables were expressed as means along with the standard deviation, minimum, and maximum values. Qualitative variables were expressed as absolute frequencies and as percentages.

The chi-square test was applied to verify whether an association existed between qualitative variables, and analysis of variance was applied to compare continuous variables between groups. *p* values below 0.05 were considered significant.

In order to analyse the relationship between PAPP-A and foetal birth weight, as well as consider the gestational age.

First, different distributions were adjusted for these variables by applying GAMLSS models (generalised additive models for location scale and shape), making it possible to evaluate not only the mean but also other parameters relating to the distribution, such as variance, kurtosis, and asymmetry, as a function of explanatory variables. Goodness of fit for the models was evaluated using the Bayesian Information Criteria as well as graphically using Q-Q plots. Finally, the best-fit distributions were selected, as well as the variables influencing the weight of the newborn and relating to gestational age.

**Fig. 1:**
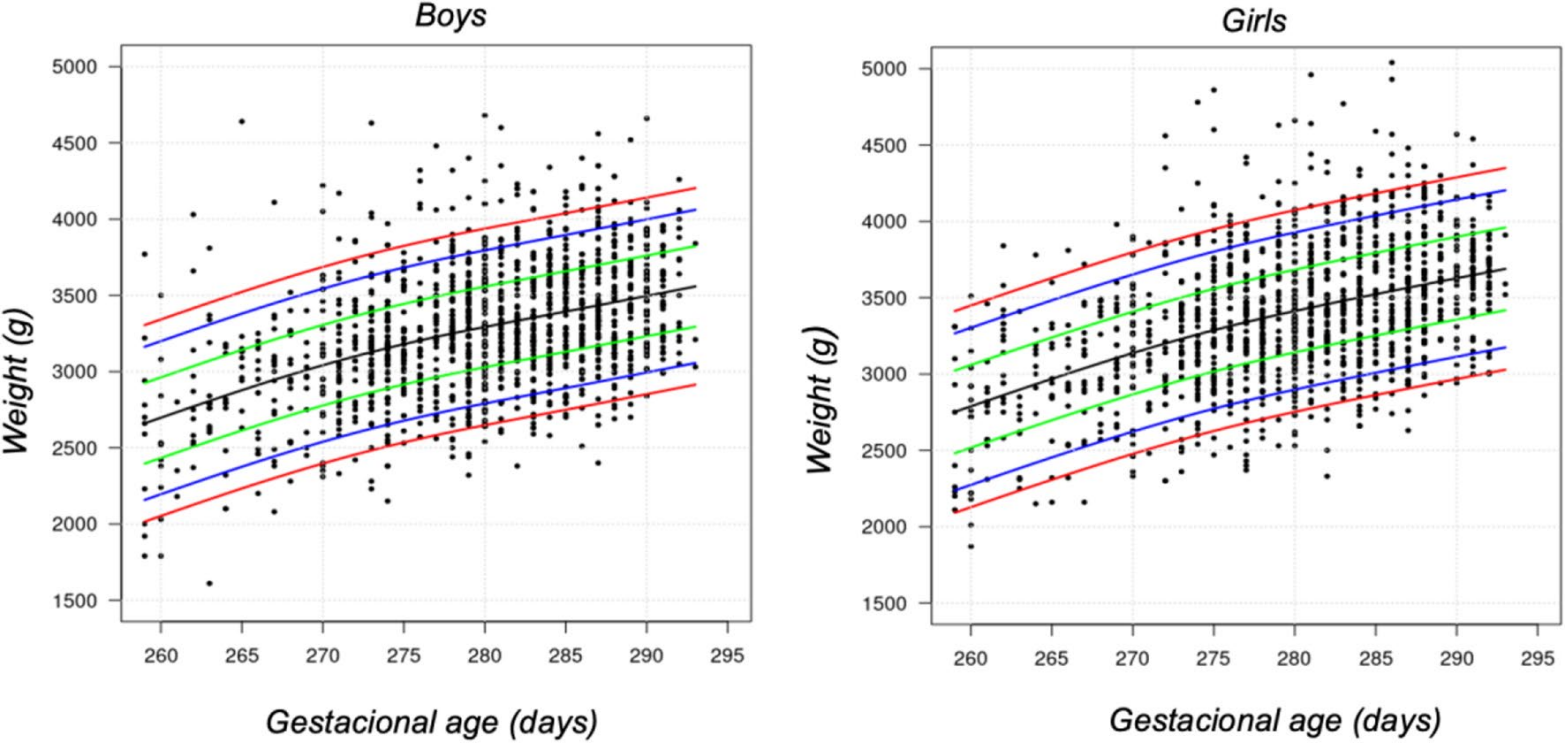
Weight percentile according to gestational age and sex

Subsequently, *Z*-score percentiles were compiled for the screening parameters as a function of the variables of newborn weight and gestational age, based on the GAMLSS regression models. In order to facilitate clinical use, different cut-off points were defined for the 5th, 10th, 25th, 50th, 75th, 90th, and 95th percentiles. In order to carry out the analysis, the “gamlss” package of the R program was used (R statistical software environment, version 4.0.1; R Foundation [11]) (Fig. 1).

From these percentiles, we established 4 groups:

- VERY LOW PAPP-A: PAPP-A value below p2 for GA
- LOW PAPP-A: PAPP-A value between p2 and p5 for GA
- NORMAL PAPP-A: PAPP-A value between p5 and p95 for GA
- HIGH PAPP-A: PAPP-A value above p95 for GA

Serum PAPP-A levels were converted to multiples-of-the-median values (MoM) by correcting for.gestational age, ethnicity, smoking status, maternal weight, and conception method, using an automated chemiluminescent immunometric assay (IMMULITE2000® Siemens).

## Results

During this period, there were 5273 births at our centre. Of these, 4970 births were from singleton pregnancies; 294 were from twin pregnancies; and 9 births were from triplet pregnancies. Of the 4970 women with singleton pregnancies, full study data could not be obtained for 734 women. Of the remaining 4236 women, 2794 of them consented to participate in the study by signing the informed consent form.

The values for PAPP-A were evaluated at the time they were determined, excluding any measurements that had been performed beyond week 13 + 6 (97 days) and those relating to preterm deliveries (< 259 days), thus reducing the sample size to 2487.

### Description of the Study Sample

In the 2794 pregnant women who participated in the study, the mean age was 32.89 years. The majority race was Caucasian (97.6%). The mean BMI was 24.82 at the beginning of pregnancy, and the mean weight gain that occurred was 12.8 kg. A total of 46% of the pregnant women were in their first pregnancy; in contrast, 36.7% of the pregnant women were second-born. The majority were healthy women (80.9% with no medical history of interest). Regarding pathologies that appeared during the current pregnancy, 20.9% of the pregnant women presented some complication during the pregnancy, such as preeclampsia or endocrinopathies (diabetes or gestational hypothyroidism). The mean length of gestation was 277.9 days.

In this period, 49.8% of women and 50.2% of men were born. The mean weight of the newborns was 3322 g. A total of 64.5% of births were spontaneous deliveries. There were 18.9% caesarean sections and 16.7% instrumental deliveries.

### Results of Relationship Between PAPP-A and Foetal Weight at Birth

**Fig. 2:**
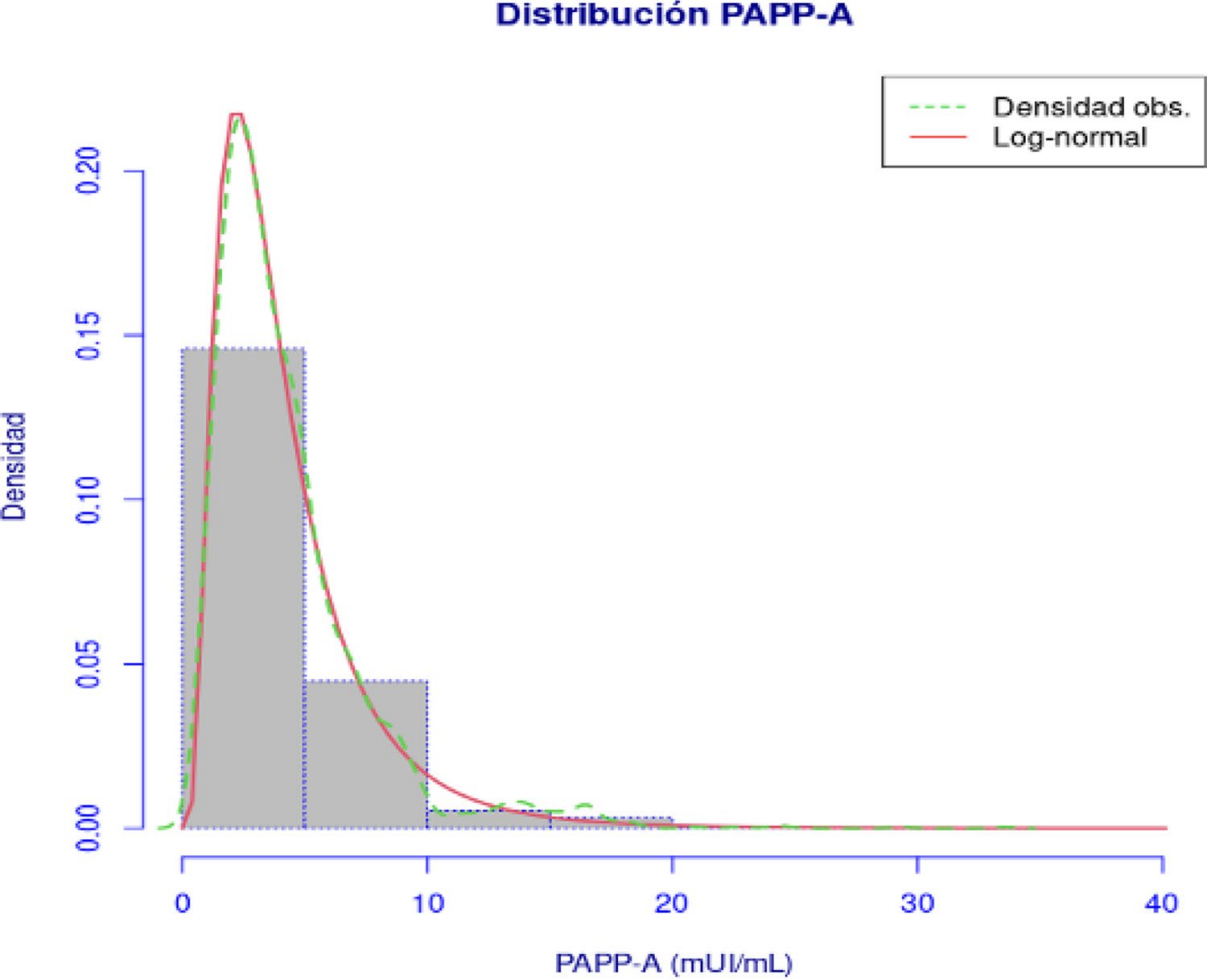
Distribution and *f*. Density for PAPP-A

The minimum value obtained was 0.32 mIU/mL, and the maximum was 33.5 mIU/mL, with a mean value of 4.25 mIU/mL and a median of 3.41 mIU/mL (Fig. 2).

The minimum value of MoM PAPP-A for our population was 0.14; the maximum value was 8.55; the mean was 1.04.

We also established 4 patient groups according to the value of the MoM PAPP-A (Table 1).

**Table 1:** Group classification according to MoM PAPP-A value

Group	No. of cases
Very low MoM PAPP-A	42 (1.78%)
Low MoM PAPP-A	189 (8%)
Normal MoM PAPP-A	1893 (80.14%)
High MoM PAPP-A	238 (10.08%)

**Fig. 3:**
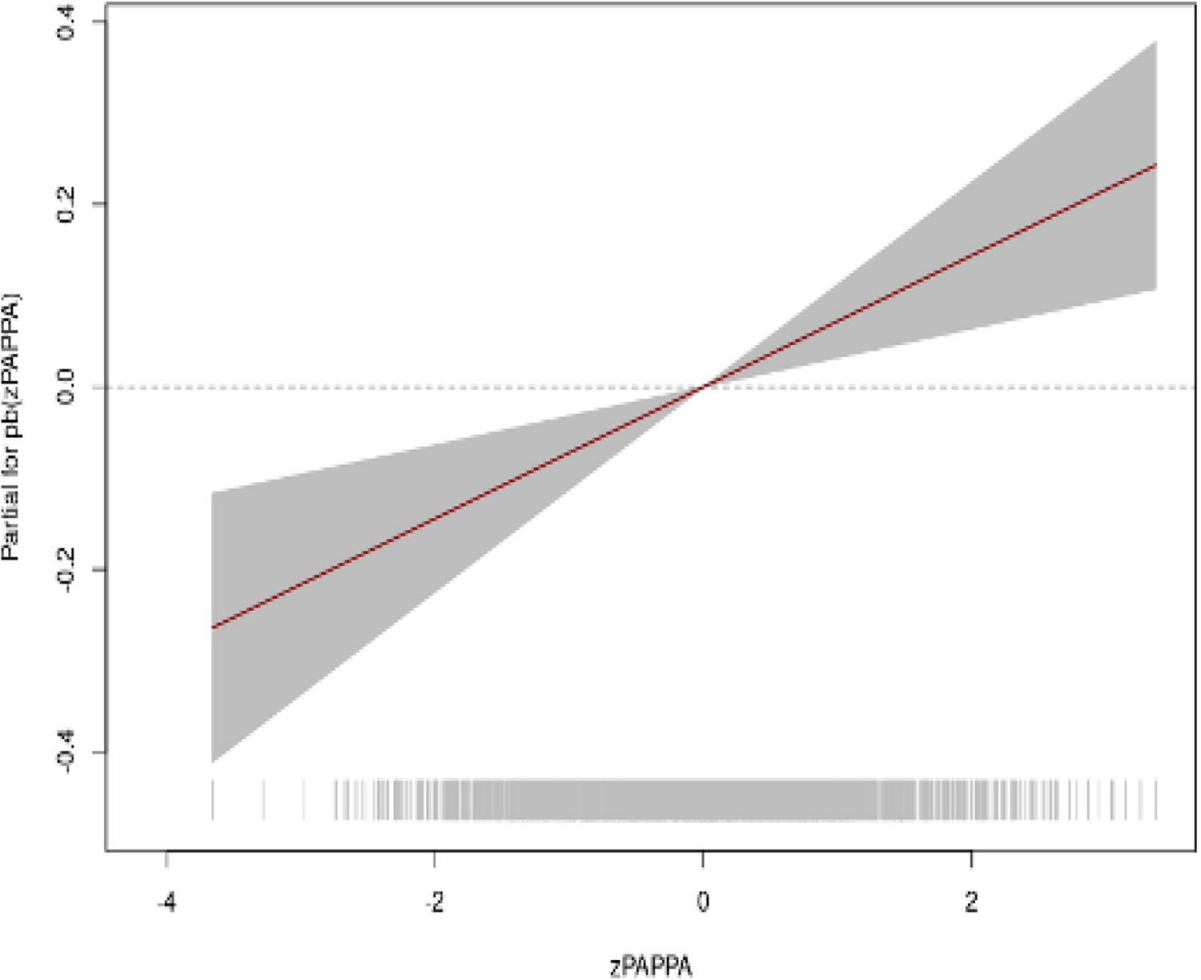
Effect of zPAPP-A on zFW (foetal weight)

On analysis of the relationship between foetal birth weight (adjusted for sex and GA) and the zPAPP-A value, we observed that the relationship is significant (*p* = 0.0003) and that it is both linear and increasing, although it shows greater variability at the extremes due to the small number of patients with extreme zPAPP-A values (Fig. 3).

**Fig. 4:**
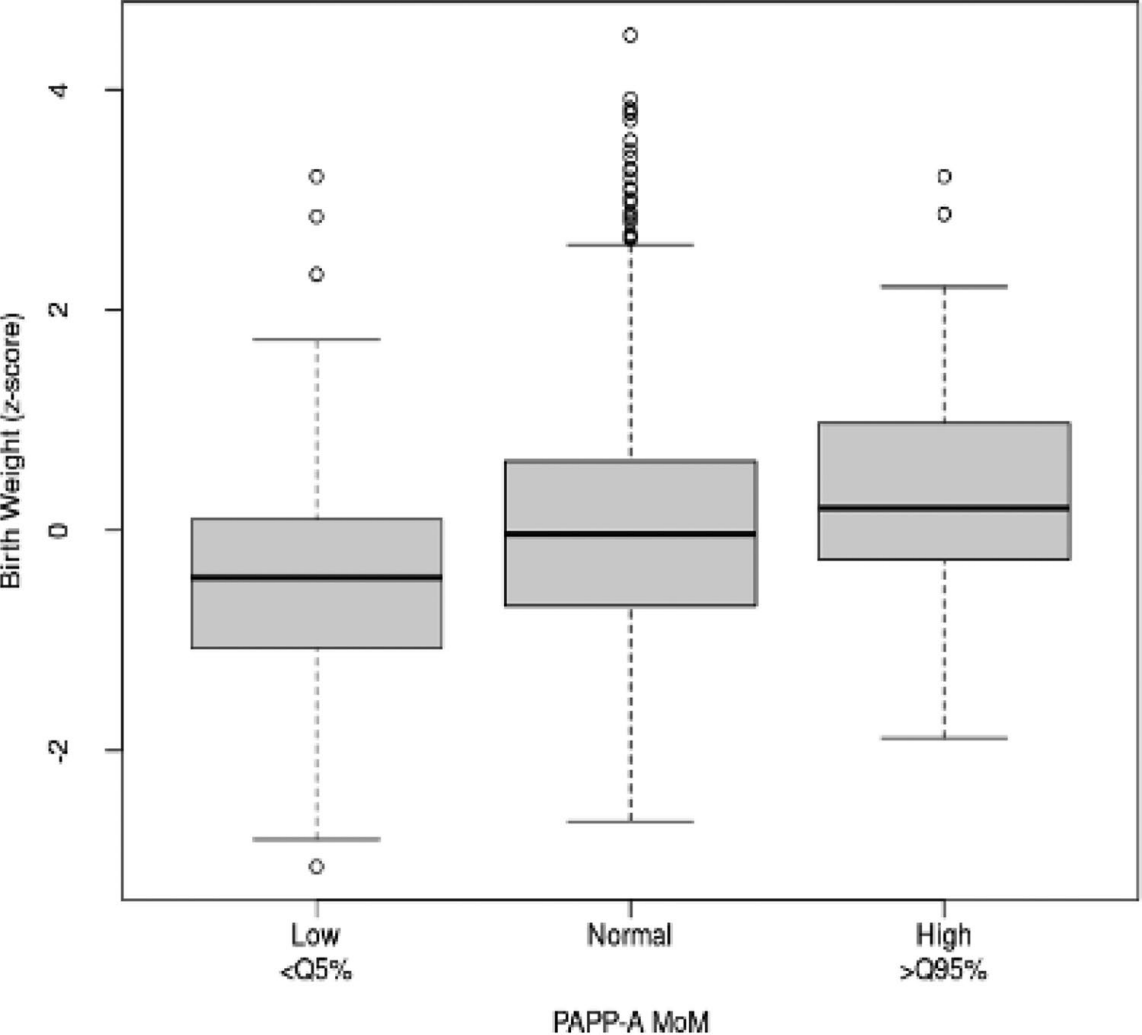
Boxplot for zFW by MoM PAPP-A group

When we considered the MoM PAPP-A groups (< p5, p5–95, and > p95) and analysed their relationship with birth weight taking into account gestational age and foetal sex, we found that there were significant differences between the three groups: the differences are particularly significant between the MoM PAPP-A < p5 and normal groups (*p* = 0.00003); and between the MoM PAPP-A < p5 and > p95 groups (*p* = 0.0000001) (Fig. 4).

The OR for low birth weight in women with very low MoM PAPP-A (< p2, corresponding to 0.3) was 2.74 (95% CI, 1.32–5.6); for low MoM PAPP-A, it was 1.52 (95% CI, 0.99–2.33); and for high MoM PAPP-A, it was 0.53 (95% CI, 0.29–0.94).

## Discussion

Examining foetal growth and the predictive variables that may have an influence on it represents a vital element for planning obstetric care. Additionally, foetal growth is believed to be an important determinant of adult health. Multiple studies have described an association between low birth weight and increased risk of an individual suffering from a wide variety of diseases in adult life, particularly ischaemic heart disease, hypertension, and metabolic diseases such as type-2 diabetes [19–21]. Since 1981, it has been possible to find a great many publications in the literature that describe relationships between a variation in PAPP-A levels and foetal complications. The first study of this kind, identifying an association between low PAPP-A levels and hypertensive gestational states, was carried out by Toopk et al. [27]. In modern times, this relationship has become a clinical reality and is applied in many hospitals as part of the protocol for predicting preeclampsia where PAPP-A values are less than 0.4 multiples of the median (MoM).

There are several publications in the literature that have reported on the laboratory markers and ultrasound features of the first trimester of pregnancy and adverse events occurring during pregnancy [32]. In 2004, the FASTER study [33], was published, which examined data from 34,271 pregnancies. In this study, associations were investigated between PAPP-A levels below p5 and abortion, RIG, preeclampsia, gestational hypertension, preterm delivery, intrauterine death, preterm premature rupture of membranes, and abruption of the normally-inserted placenta [34]. A meta-analysis by Morris et al. on this subject included 32 studies and 175,240 pregnancies. It concluded that there is a moderate association between PAPP-A < p5 and adverse events in pregnancy, and if we consider data for < p1, then this association becomes strong [35]. This result is in line with results seen in our population: despite cases having MoM PAPP-A at very low levels (below 0.3) being associated with almost triple the probability of having a foetus with low weight for gestational age, most women with MoM PAPP-A below this level still gave birth to children between p10 and p90, which therefore were considered foetuses of normal weight. These data confirm that PAPP-A may be a predictor of foetal birth weight given that the risk of having a foetus weighing below p10 decreases as the MoM PAPP-A value increases.

The relationship between increased PAPP-A and macrosomia is also reflected in the literature. One study that included 1,371 cases found that when PAPP-A is > p90, the adjusted OR for macrosomia is 2.9 [36]. A Spanish study including 121 pregnant women found that PAPP-A values expressed as MoM behave as an independent predictor of foetal macrosomia, independently of maternal factors or parameters seen on early foetal ultrasound. As such, a MoM PAPP-A value above 1.89 can predict foetal macrosomia with a specificity of greater than 80% [37]. This relationship cannot, however, be seen in most large studies. The FASTER study found a relationship between macrosomia and PAPP-A > p95, but this was not statistically significant [33]. The authors considered that there is a negative relationship between low levels of PAPP-A and the risk of macrosomia.

In this study, we found that the probability of having an overweight new-born increased with MoM PAPP-A in a linear fashion up to around 1.25 and that this trend was no longer observed beyond this value, with the probability existing of having a macrosomic new-born (*p* = 0.0081). In other words, for MoM PAPP-A values lower than ~ 1.25, the greater the MoM PAPP-A, the greater the probability of falling into the overweight group; while for values greater than 1.25, the MoM PAPP-A had no effect on this probability. Moreover, as seen in the FASTER study, women with very low MoM PAPP-A values in the first trimester (< 0.3) have a very low probability of having an LGA new-born (2.38% vs 9.93% when the MoM PAPP-A value is normal) [33]. In our study, the value of MOM PAPP-A levels as a predictor of foetal macrosomia, a correlation could be observed with elevated levels, although this was not statistically significant. In contrast, Poon et al. has shown the relation between birth weight centile and PAPP-A is statistically significant, and the first-trimester serum levels are increased in pregnancies delivering macrosomic neonates [38].

We can therefore see that extreme values of MoM PAPP-A can act as early predictors of impaired foetal growth. According to our results, it is important to pay particular attention in cases of pregnant women who have MoM PAPP-A values of less than 0.3 in the first trimester, as the risk of foetal growth below p10 is almost three times the rate seen in those with normal MoM PAPP-A values at first-trimester combined screening.

## Conclusions

MoM PAPP-A values determined during the first-trimester act as a predictor of foetal weight at full-term birth. As a result, this parameter could be used as a tool to screen for growth restriction, opening the door to possible further studies into treatments that eliminate or decrease the risk of impaired foetal growth. As opposed to our centre, the utility of MoM PAPPA as a predictor of macrosomia was not statistically significant (*p* = 0.204).
